# Natural Bioactives and Phytochemicals Serve in Cancer Treatment and Prevention

**DOI:** 10.1155/2013/698190

**Published:** 2013-12-19

**Authors:** Shun-Fa Yang, Chia-Jui Weng, Gautam Sethi, Dan-Ning Hu

**Affiliations:** ^1^Institute of Medicine, Chung Shan Medical University, Taichung 402, Taiwan; ^2^Department of Medical Research, Chung Shan Medical University Hospital, Taichung 402, Taiwan; ^3^Graduate Institute of Applied Living Science, Tainan University of Technology, Tainan City 710, Taiwan; ^4^Department of Pharmacology, Yong Loo Lin School of Medicine, National University of Singapore, Singapore 119077; ^5^Tissue Culture Center, New York Eye and Ear Infirmary, New York Medical College, New York, NY 10009, USA

Natural bioactives or phytochemicals are generally referred to as the compounds that have specific biological activity in human. Studies have shown that natural phytochemicals derived from certain plants have the capability to prevent carcinogenesis. In this special issue, we collected numerous studies which provide novel evidence to support the opinion. For instance, [6]-gingerol prevents disassembly of cell junctions and activities of MMPs in invasive human pancreas cancer cells; butein inhibits angiogenesis of human endothelial progenitor cells; the anticancer effects of sterol fraction come from red algae porphyra dentata; zeaxanthin induces apoptosis in human uveal melanoma cells; and ocotillol enhanced the antitumor activity of doxorubicin. It is therefore believed that the appropriate application of natural bioactives or phytochemicals should be a supplementary and safe way to prevent carcinogenesis and/or to enhance the efficacy of cancer therapy.

*Shun-Fa  Yang* *Shun-Fa  Yang* *Chia-Jui  Weng* *Chia-Jui  Weng* *Gautam  Sethi* *Gautam  Sethi* *Dan-Ning  Hu* *Dan-Ning  Hu*

